# Noncoding RNAs in Glioblastoma: Emerging Biological Concepts and Potential Therapeutic Implications

**DOI:** 10.3390/cancers13071555

**Published:** 2021-03-28

**Authors:** Uswa Shahzad, Stacey Krumholtz, James T. Rutka, Sunit Das

**Affiliations:** 1 Institute of Medical Science, Faculty of Medicine, University of Toronto, 1 King’s College Circle, Medical Sciences Building, Toronto, ON M5S 1A8, Canada; uswa.shahzad@utoronto.ca (U.S.); james.rutka@sickkids.ca (J.T.R.); 2 Arthur and Sonia Labatt Brain Tumor Research Center, Hospital for Sick Children, 686 Bay Street, Toronto, ON M5G 0A4, Canada; stacey.krumholtz@sickkids.ca; 3 Division of Neurosurgery, St. Michael’s Hospital, 30 Bond Street, Toronto, ON M5B 1W8, Canada

**Keywords:** microRNA, long noncoding RNA, circRNA, piRNA, GBM

## Abstract

**Simple Summary:**

Since the completion of the Human Genome Project, noncoding RNAs (ncRNAs) have emerged as an important class of genetic regulators. Several classes of ncRNAs, which include microRNAs (miRNAs), long noncoding RNAs (lncRNAs), circular RNAs (circRNAs), and piwi-interacting RNAs (piRNAs), have been shown to play important roles in controlling developmental and disease processes. In this article, we discuss the potential roles of ncRNAs in regulating glioblastoma (GBM) formation and progression as well as potential strategies to exploit the diagnostic and therapeutic potential of ncRNAs in GBM.

**Abstract:**

Noncoding RNAs (ncRNAs) have emerged as a novel class of genomic regulators, ushering in a new era in molecular biology. With the advent of advanced genetic sequencing technology, several different classes of ncRNAs have been uncovered, including microRNAs (miRNAs), long noncoding RNAs (lncRNAs), circular RNAs (circRNAs), and piwi-interacting RNAs (piRNAs), which have been linked to many important developmental and disease processes and are being pursued as clinical and therapeutic targets. Molecular phenotyping studies of glioblastoma (GBM), the most common and lethal cancer of the adult brain, revealed that several ncRNAs are frequently dysregulated in its pathogenesis. Additionally, ncRNAs regulate many important aspects of glioma biology including tumour cell proliferation, migration, invasion, apoptosis, angiogenesis, and self-renewal. Here, we present an overview of the biogenesis of the different classes of ncRNAs, discuss their biological roles, as well as their relevance to gliomagenesis. We conclude by discussing potential approaches to therapeutically target the ncRNAs in clinic.

## 1. The Noncoding Genome—Challenging the Central Dogma

The central dogma of molecular biology posits that genetic information is passed from DNA → RNA → protein. Over the past two decades, the advent of high-throughput genomic sequencing technology has challenged the previously held view of transcriptome as a mere messenger between the genome and the proteome. The RNAs are divided into two distinct classes: messenger RNAs (mRNAs), which are translated into proteins, and the non-protein coding RNAs (ncRNAs) [1]. The ncRNA transcripts that are shorter than 200 bp in length are classified as small noncoding RNAs, which include transfer RNAs (tRNAs), small nucleolar RNAs (snoRNAs), small nuclear RNAs (snRNAs), microRNAs (miRNAs), and piwi-interacting RNAs (piRNAs), among others. Long noncoding RNAs (lncRNAs) are described as transcripts that are longer than 200 nucleotides and have no protein coding potential. Certain classes of circular RNAs (circRNAs) also fall under the definition of lncRNAs [2,3,4,5].

Even though ncRNAs do not encode for proteins, they hold important biological information and play a variety of roles in cell fate, development, and disease. Recent studies have implicated their role in regulating progression and pathogenesis of glioblastoma (GBM) [6,7] and, in particular, in glioma cancer stem cells (GSCs), which are thought to be resistant to chemotherapy and radiation and drive tumor recurrence [8,9,10].

In this paper, we provide an overview of four different species of ncRNAs by discussing their biogenesis and functions. Additionally, we highlight key ncRNAs and explore their potential as putative therapeutic targets in glioma biology. We also discuss various strategies to exploit their diagnostic and therapeutic potential, which may serve as a valuable introductory tool for the study of ncRNAs in GBM.

## 2. MicroRNAs—Small, but Powerful Gene Regulatory Machines

MicroRNAs are perhaps the most well-studied and widely characterized class of ncRNAs. They are short, endogenous RNAs between 17–22 nucleotides long that post-transcriptionally regulate gene expression and mediate gene silencing. In 1993, Victor Ambros and colleagues discovered the first miRNA in *Caenorhabditis elegans* (*C. elegans*), when they described a small RNA encoded by the lin-4 locus that plays a crucial role in controlling temporal identities of cells during postembryonic developmental events in the worm [11,12,13]. Since their initial discovery, a total of 48,860 mature miRNAs have been discovered in 271 different organisms [14]. In addition, in humans, approximately 1917 hairpin precursors and at least 2654 mature miRNAs have been annotated [14]. Around half (46%) of all miRNAs in humans are intragenic and are processed from the introns, and relatively few from exons, of protein-coding transcripts. The remainder (54%) are produced independently from intergenic non-coding pri-miRNA transcripts and are regulated by their own promoters [15,16]. Without a doubt, miRNAs comprise one of the more abundant classes of gene regulatory molecules and are critical for normal development due to the fact of their ability to influence the expression of protein-coding genes. Therefore, unsurprisingly, their aberrant expression has been linked to several disease processes.

### 2.1. Dynamics of MicroRNA-Mediated Gene Regulation

**Figure 1:**
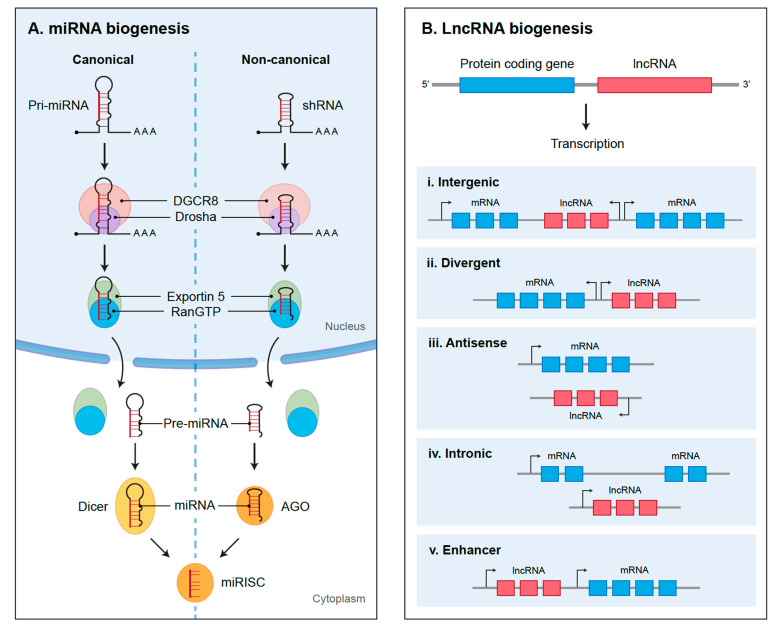
The biogenesis of noncoding RNAs. (**A**) The biogenesis of microRNAs (miRNAs) can occur through either the dominant canonical pathway or the non-canonical pathway. In the canonical pathway, piwi-interacting RNAs (pri-miRNAs) are cleaved by the microprocessor complex, composed of DGCR8 and Drosha, into hairpin structures called the pre-miRNA. The pre-miRNA is exported by the Exportin5/RanGTP complex to the cytoplasm, where it is further cleaved by Dicer into ~22 long nucleotide mature miRNA duplexes. In contrast, the short hairpin RNA (shRNA) transcripts use the Dicer-independent pathway. Here, they are initially cleaved by the microprocessor and exported to the cytoplasm via the Exportin5/RanGTP complex and further cleaved by the Argonaute (AGO) to produce a mature miRNA. Regardless of whether an miRNA is generated through a canonical or non-canonical process, all pathways ultimately lead to a functional miRNA-induced silencing complex (miRISC) that is composed of a guide strand and AGO. (**B**) Long noncoding RNAs (LncRNAs) are transcribed from their respective locations on the genome and are often defined by their location relative to the neighbouring protein-coding genes. Intergenic lncRNAs are transcribed from loci in between two protein-coding genes, whereas intronic are transcribed from inside of an intron of a protein-coding gene. The bidirectional lncRNAs are products of divergent transcription from the promoter of a protein-coding gene. Antisense lncRNAs, on the other hand, initiate inside of a 3’ end of a protein-coding gene and transcribed in the opposite direction.

The biogenesis of miRNAs can occur through either the main canonical pathway or the non-canonical pathway (Figure 1A). In the canonical pathway, pri-miRNAs are transcribed from their respective genes by RNA polymerase II [17]. The pri-miRNA is then capped, polyadenylated, and folded into a stem–loop structure via intermolecular base paring [17]. The microprocessor complex, composed of an RNA-binding protein, called DiGeorge Syndrome Critical Region 8 (DGCR8; also known as Pasha), and a ribonuclease III enzyme, named Drosha, then cleaves each pri-miRNA into 60–120 nucleotide long hairpin structures called the pre-miRNA [18,19]. Next, the pre-miRNA is exported by the Exportin5/RanGTP complex from the nucleus to the cytoplasm, where it encounters the RNase III enzyme Dicer, which further cleaves it into ~22 long nucleotide mature miRNA duplexes [18,20]. The miRNA duplex is then unwound, and either the 5p strand of the miRNA, which originates from the 5’ end of the pre-miRNA hairpin, or the 3p strand, which arises from the 3’ end, is selected by Argonaute (AGO) and loaded into the RNA-induced silencing complex (RISC). The miRNA leads RISC to its complementary mRNA molecule, resulting in degradation or translational inhibition of the target mRNA [21,22].

In comparison, the non-canonical miRNA biogenesis pathways can be classified into Drosha/DGCR8-independent or Dicer-independent pathways. The Drosha/DGCR8 independent pathway is utilized by pre-miRNAs that resemble Dicer substrates that include the 7-methylguanosine (m7G)-capped pre-miRNAs as well as mirtrons that are transcribed from introns of protein-coding genes during splicing events. These pre-miRNAs are exported directly to the cytoplasm by Exportin 1, without the need to undergo cleavage by Drosha [23,24,25,26]. In contrast, short hairpin RNA (shRNA) transcripts use the Dicer-independent pathway. Here, the pre-miRNA transcripts are initially cleaved by the microprocessor and exported to the cytoplasm via the Exportin5/RanGTP complex. They are then loaded into AGO2 and are cleaved by the AGO catalytic center to generate an intermediate 3’ end, which is then further trimmed to produce a mature miRNA [27,28].

Regardless of whether a miRNA is generated through a canonical or non-canonical process, all pathways ultimately lead to a functional miRNA-induced silencing complex (miRISC) that is composed of a guide strand and a member of the AGO protein family. The miRISC gains target specificity by recognizing the complementary miRNA response elements (MREs) found on the target mRNA [29,30]. Mammalian miRNAs can have an average of 300 conserved target mRNAs per miRNA family [31]. The complementary miRNA:MRE interaction primarily occurs through recognition of a “seed region” that consists of nucleotides 2–8 from the 5’ end of the miRNA [32]. This also poses a challenge as most target prediction algorithms rely on searching for the presence of conserved sequences of 6, 7, or 8 nucleotides that match the seed region of each miRNA, generating hundreds of predicted target matches [33].

Moreover, the target mRNA cleavage requires extensive sequence complementarity, because it determines whether there is AGO2-dependent slicing of the target mRNA or miRISC-mediated translation inhibition and target mRNA decay [34]. Hence, when a fully complementary miRNA:MRE interaction is established, it induces AGO2 endonuclease cleavage which, incidentally, also destabilizes the 3’ end of the guide miRNA, consequently resulting in its degradation as well [29,35,36]. In humans, miRNAs rarely form exact matches to the target mRNAs they regulate. In fact, the majority of the MREs contain at least central mismatches, which makes it extremely unlikely that the target mRNAs will undergo AGO2 endonuclease activity and that the 3′ end of miRNA is destabilized [35,36].

The miRISC complex additionally recruits the GW182 family of proteins, which acts as a scaffold for recruiting more effector proteins that are involved in poly (A) deadenylation and the mRNA decapping processes [29,37]. Finally, the decapped and deadenylated mRNA strands are degraded by the 5′-3′ exoribonuclease 1 (XRN1) [30,38,39].

While miRNAs are most commonly associated with translational repression and gene silencing, more evidence has emerged that they may also mediate transcriptional and translational activation [40,41]. For example, in serum-starved cells, AGO2 forms a complex with Fragile-x mental retardation related protein 1 (FXR1), instead of GW182, and the resulting complex associates with AU-rich elements (AREs) at 3′UTR to activate translation [37]. The miRNA-mediated gene upregulation has also been observed in quiescent cells like oocytes [42]. This suggests that miRNAs have a more diverse biological role within gene regulatory networks.

The dynamics of any given miRNA within the cell are highly dependent on the total number of available binding sites for it on all targetable RNA molecules. While a particular miRNA may interact with most or all of the available MREs, the RNAs with higher affinity MREs will retain it longer, with the result of greater transcriptional repression. The binding affinity of a miRNA is mainly dependent upon the extent of miRNA seed base pairing, mismatches, as well as the secondary structure of the target RNA [43,44,45]. Since only a small proportion of each target mRNA may be bound to its complementary miRNA at any given time, this may also result in a dosage dilution effect [46]. The capacity of a miRNA to bind to available MREs may be further affected by competing endogenous RNAs (ceRNAs) that contain complementary MRE sites for it, such as circRNAs or lncRNAs [47,48,49,50]. The ceRNAs may sequester an miRNA away from its target mRNA, preventing the transcriptional repression of the target mRNA [44,47]. An example of a ceRNA is highly upregulated in liver cancer (HULC), a well-characterized cancer-associated lncRNA that is a potential biomarker for hepatocellular carcinoma (HCC) [47,48,49,50]. HULC acts as a ceRNA by binding to miR-372 and suppressing the translational inhibition of miR-372 target genes [51]. In contrast, an individual mRNA may have many MREs for binding different miRNAs, which can enhance effects on gene regulation through the cooperative efforts of several miRNAs. For example, cyclin-dependent kinase inhibitor 1A (CDKN1A, also known as p21Cip1/Waf1), a tumour-suppressor downregulated in many cancers, is targeted by at least 28 miRNAs. Many of these miRNAs are upregulated in cancers with altered expression of CDKN1A, suggesting a synergistic effect of miRNAs [51].

Besides MRE load, much of the miRNA-mediated gene regulation is reliant upon the subcellular compartmentalization of the miRISC complex. The miRISC has been observed in the nucleus, mitochondria, rough endoplasmic reticulum (rER), trans-Golgi network, lysosomes, stress granules, processing (P)-bodies, and endosomes [52]. In the nucleus, miRISC can either associate with DNA to promote active or inactive chromatin states or interact with nascent mRNA to influence alternative splicing. In the cytoplasm, including the mitochondria, miRISC can mediate mRNA decay, inhibit translation initiation, or promote translational activation. The miRISC can additionally inhibit translation by interacting with translating mRNA in the rough ER. The miRISC can also be stored in stress granules or shuttled to lysosomes for degradation. Finally, miRISC may be secreted via exosomes to the other cells to mediate cell–cell communication and may be internalized via endosomes through the Golgi [53,54,55,56,57,58,59,60,61].

It is evident that miRNAs have dynamic roles in gene regulation, as they not only mediate mRNA stability but also the integral processes of transcription and translation. Hence, they are important regulatory molecules that help control the development and progression of human tumours.

### 2.2. miRNAs in Gliomas

**Figure 2:**
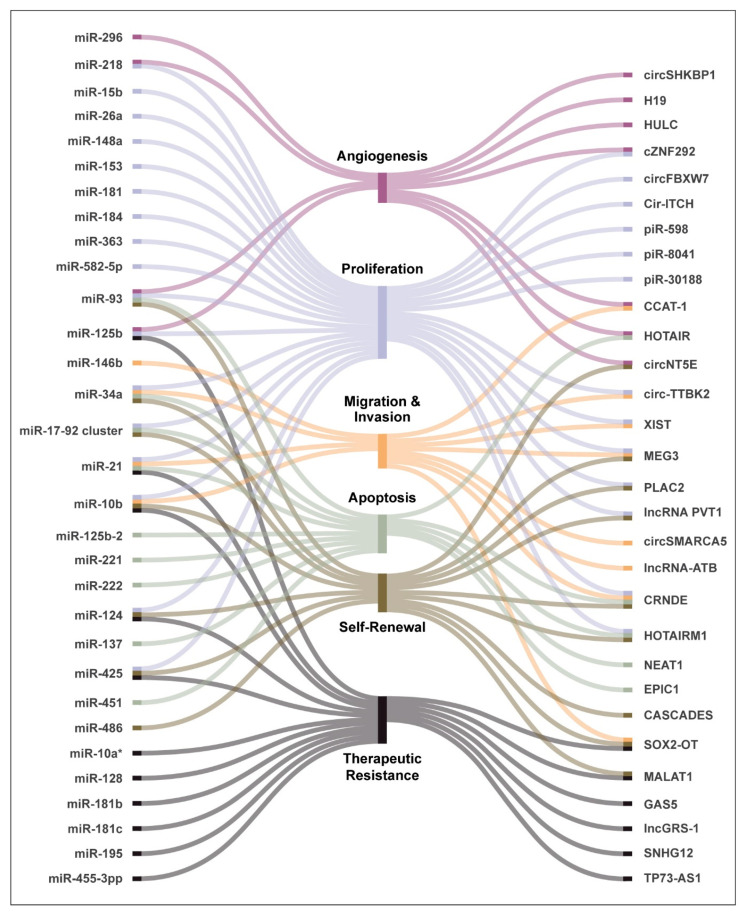
NcRNAs and regulation of hallmarks of GBM. Noncoding RNAs can regulate several hallmark processes associated with gliomas including controlling cellular proliferation and apoptosis, regulating migration and invasion potential of the tumor cells, modulating therapeutic response, self-renewal, and angiogenesis.

**Figure 3:**
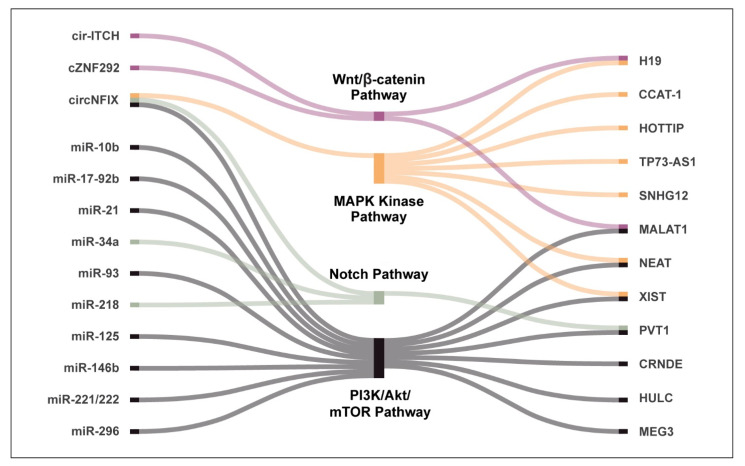
NcRNAs and key GBM pathways. Noncoding RNAs can interact with or engage in crosstalk with several key pathways that are dysregulated in gliomas including the PI3K/Akt/mTOR pathway, Notch signaling, and Wnt/β-catenin pathway.

Due to their complex nature, miRNAs can act as either oncogenes or tumor suppressors through regulation of other protein-coding genes. Several miRNAs have been shown to be dysregulated in GBM and are associated with poor survival (Table 1) [53,54,55,56,57,58,59,60,61]. A recent systematic review by Moller and colleagues reported that 256 miRNAs (including miR-21, miR-17, miR-93, and miR-221/222) were significantly upregulated, whereas another 95 (notably miR-7, miR-34a, miR-128, and miR-137) were downregulated in GBM compared to normal brain [62]. The miRNAs have been shown to modulate many hallmark characteristics of GBM, including cell proliferation, migration and invasion, self-renewal, angiogenesis, and therapeutic resistance, among others (Figure 2). Additionally, since they can be excreted via vesicles, they can also be used as biomarkers [63]. Here, we discuss some of the well-characterized miRNAs that have been implicated in regulating key pathways in glioma biology (Figure 3).

#### 2.2.1. Cell Proliferation and Apoptosis

One of the first miRNAs to be linked with glioma malignancy was miR-21, which is highly upregulated in GBM and has expression levels that correlate with WHO tumour grade [113]. miR-21 has been shown to target tumour suppressor genes, such as PDCD4 (programmed cell death 4), ANP32A (acidic nuclear phosphoprotein 32 family member A), SMARCA4 (SWI/SNF related, matrix-associated, actin-dependent regulator of chromatin, subfamily A, member 4), PTEN (phosphatase and tensin homolog), and SPRY2 (Sprouty RTK signaling antagonist 2), and its inhibition leads to significantly reduced tumour cell proliferation and decreased tumour growth in immunodeficient mice [62,65,70,71,72,73,74,75,76,77,78]. In addition, miR-21 exerts antiapoptotic effects by inhibiting HNPRK (heterogenous nuclear ribonucleoprotein K), Tap63 (tumour suppressor homologue of p53), and PDCD4 [77,114,115,116,117].

miR-221/222 is also upregulated in GBM and targets the tumour suppressor p27 and p53-upregulated modulator of apoptosis (PUMA), which can induce cell death via binding to Bcl-2 and Bcl-xL. Overexpression of miR-221/222 can suppress the expression of PUMA, inhibiting apoptosis and promoting cell survival [77,117,118,119].

miR-34a is a significantly downregulated miRNA in GBM and has been shown to directly inhibit the expression of receptor tyrosine kinases and critical regulators of cell fate, such as MET, NOTCH1 and 2, CDK6, CCND1, and SIRT1, through which it directly regulates cell proliferation and survival as well as self-renewal and cell invasion in GBM [87,88,89,90]. Several other miRNAs, including miR-15b, miR-26a, miR-125b, miR-148a, miR-153, miR-181, miR-184, miR-1218, miR-363, and miR-582-5p, have been reported to regulate cell viability and proliferation and promote tumour growth [79,80,81].

#### 2.2.2. Migration and Invasion

In addition to its role in proliferation and cell survival, miR-21 has been implicated in regulating GBM cell migration and invasion potential by suppressing matrix remodeling proteins that normally regulate the levels of matrix metalloproteinases (MMPs) such as TIMP3 (tissue inhibitor of metalloproteinases 3) and RECK (reversion inducing cysteine rich protein with Kazal motifs), ANP32A, and SPRY2 [63,113]. miR-146b has also been reported to inhibit MMP16 and enhance GBM cell invasion [120,121]. In a similar manner, miR-10b promotes cell invasion by targeting HOXD10, resulting in increased MMP14 expression [84].

#### 2.2.3. Angiogenesis

Several miRNAs, termed “angiomiRs”, have been shown to play important roles in supporting and regulating the vascular network in GBM. One such angiomiR, miR-296, is highly upregulated in endothelial cells stimulated with pro-angiogenic growth factors, such as vascular endothelial growth factor (VEGF), or in the presence of glioma cells. Augmented expression of miR-296 has been associated with increased endothelial cell tube formation as well as enhanced tumour vascularization. Knockdown of miR-296 attenuates tumour angiogenesis [75,94,95].

miR-125b is an angiomiR that is significantly downregulated in GBM-associated endothelial cells and targets myc-associated zinc finger protein (MAZ), a transcription factor that regulates VEGF. In the case of miR-125b repression, MAZ is overexpressed, which in turn promotes tumour vascularization [91]. On the other hand, miR-218 was reported to target the hypoxia-inducible factor, HIF-2α, and thereby attenuate GBM tumour neovascularization and preventing cell survival [83]. miR-93, which is a member of the miR-17 family, is another angiomiR that is upregulated in GBM. miR-93 enhances cell survival, sphere formation and induces blood vessel formation through suppression of integrin-β8. In a report by Fang et al. [86], overexpression of miR-93 in GBM cells led to enhanced endothelial cell proliferation and tube formation in vitro, as well as increased vasculogenesis in mouse xenografts.

#### 2.2.4. Therapeutic Resistance

MicroRNAs can influence therapeutic response to chemoradiation and can also contribute to drug resistance. For example, miR-21 reduces the efficacy of the cytotoxic effect of temozolomide (TMZ) by upregulating the antiapoptotic protein Bcl-2 and inhibiting pro-apoptotic proteins like Bax and caspase-3 [82]. More excitingly, inhibition of miR-21 using antisense-miR-21 was shown to increase the GBM cell line sensitivity to radiotherapy and chemotherapeutic drugs such as paclitaxel, sunitinib, doxorubicin, and VM-26 [122]. miR-125b-2 has also been shown to increase the resistance of GBM stem cells to TMZ. Inhibition of miR-125b-2 using peptide nucleic acid (PNA) led to enhanced cellular apoptosis in response to TMZ through activation of PARP and caspase-3 as well as release of cytochrome C from the mitochondria [122,123,124,125,126,127,128].

In a study by Ujifuku and colleagues [129], miRNA microarray analysis of a TMZ-sensitive GBM cell line revealed that miR-195, miR-455-3p, and miR-10a* were the three most upregulated miRNAs in the resistant cells. Knockdown of miR-195 enhanced the effectiveness of TMZ [85]. In a similar study, Salby et al. [85] examined the correlation between miRNAs and TMZ resistance in GBM by studying 22 primary GBM tumours and found that miR-221, miR-222, miR-181b, miR-181c, and miR-128 were significantly downregulated. Furthermore, miR-181b and miR-181c were shown to correlate with GBM response to TMZ treatment, suggesting that they can be used as predictive biomarkers for response to TMZ therapy.

#### 2.2.5. Self-Renewal

A number of miRNAs have been reported to modulate self-renewal in GSCs. In a study by Silber et al. [130], miR-124 and miR-137 were both shown to be downregulated in GBM compared to normal brain. Transfection of miR-124 and miR-137 inhibited proliferation by G1 cell cycle arrest in mouse neural stem cells and induced differentiation of GSCs. miR-34a has also been implicated in maintaining stemness of GSCs and is downregulated in human GBM [75]. Li and colleagues reported that forced overexpression of miR-34a inhibits proliferation and invasion both in vitro and in vivo in immunodeficient mice. Additionally, miR-34a was shown to sequester Notch1 mRNA, creating a threshold where a bimodal Notch signal determines the choice between self-renewal versus differentiation [131,132,133]. Further studies by the group showed that miR-34a overexpression also increased cell differentiation and apoptosis in GSCs in vitro [134]. Another miRNA profiling study reported that miR-451, miR-486, and miR-425 were upregulated in GSCs compared to differentiated GBM cells [135].

It is evident from the literature that a number of miRNAs have both oncogenic and tumor suppressive roles in GBM pathogenesis, affecting a variety of hallmarks associated with GBM such as cell migration and invasion, proliferation, apoptosis, and self-renewal, among others. Evidently, they hold great promise as potential therapeutic targets for affecting glioma initiation, development, and progression.

## 3. Long Noncoding RNAs—From “Dark Matter” to Genome Regulation

The breadth of lncRNAs was uncovered on a large scale in 2009 by Guttman and colleagues, who used chromatin-state maps coupled with massively parallel RNA-sequencing to characterize discrete transcriptional units interspersed between protein-coding loci [136,137,138]. Using this approach, they identified over a thousand highly conserved large intergenic noncoding RNAs (lincRNAs) in mammals. Many of these lincRNAs have been found to regulate diverse biological processes including cell cycle regulation, immune surveillance, and pluripotency. Furthermore, ~20% of lincRNAs are associated with chromatin-modifying complexes and have been shown to regulate chromatin conformation and affect gene expression during cell differentiation and development [139,140]. Currently, there are more than 167,000 annotated lncRNAs in humans, and the functional and biological relevance of a large majority of them still remains an enigma [141,142].

Long noncoding RNAs are often defined by their location relative to the neighbouring protein-coding genes (Figure 1B) and are classified as either intergenic (in between two protein-coding genes), intronic (transcribed from inside of an intron of a protein-coding gene), bidirectional (divergent transcription from the promoter of a protein-coding gene), or antisense (initiated inside of a 3’ end of a protein coding gene and transcribed in the opposite direction) [143]. They have been ascribed a variety of functions, including regulation of gene expression through interaction with proteins and other RNA molecules, organization of nuclear domains via paraspeckle formation, and transcriptional regulation in either cis or trans formation [144]. Much like proteins, lncRNA function is dependent on their subcellular localization. In the nucleus, lncRNAs have been shown to assemble nuclear domains via formation of paraspeckles, associate with chromatin-modifying complexes and regulate gene expression as well as transcriptional silencing, and mediate gene–gene interactions within and across chromosomes [144,145,146]. In comparison, cytoplasmic lncRNAs control gene expression by modulating mRNA translation and stability, protein localization and turnover, sponging cytoplasmic factors and regulating their availability, and scaffolding of proteins involved in a shared signaling pathway [142,147,148,149].

Undoubtedly, lncRNAs have emerged as an important class of regulators of gene expression and may play an important role in the etiology of several diseases.

### 3.1. The Diversity of Long Noncoding RNA Functions

Given the complexity of the mammalian transcriptome and the very large number of lncRNAs occupying the genome, it is not surprising that there is considerable diversity in the functions and mechanisms of lncRNAs.

Overall, the mechanisms of action through which lncRNAs exert their function can be classified under five different themes, which will be discussed below.

(1) Decoy: lncRNAs can directly bind and sequester DNA-binding proteins and other transcription factors, thus preventing them from accessing their target gene and inhibiting gene expression [150,151,152];
(2) Scaffold: lncRNAs can act as scaffolding complexes to bring two or more proteins together into close spatial proximity. In the nucleus, these scaffolds can promote selective activation or repression of gene expression based on the nature of the chromatin complex [144];
(3) Guide: lncRNAs can serve as guides and recruit proteins and chromatin-modifying complexes to specific sites in the genome [146];
(4) Sponge: the competitive endogenous RNA (or “microRNA sponge”) hypothesis suggests that lncRNAs communicate with other types of RNA through microRNA response elements. This relationship could be reciprocal and may cause the levels of one RNA to influence the activity of another. Moreover, the number of shared MREs between RNA molecules is directly related to the level of “communication” and co-regulation of those transcripts [144];
(5) Enhancer: lncRNAs can interface with chromatin-modifying complexes through chromosome looping and may engage in enhancer-based gene activation [52,53,130,132,133].

### 3.2. The “Linc” to Disease—Long Noncoding RNAs in Gliomas

Long noncoding RNAs have been implicated in glioma development through their regulation of several tumourigenic processes such as cellular proliferation, migration, and invasion as well as maintenance of GSC self-renewal and differentiation (Figure 2) [153,154]. In addition, several lncRNAs have been shown to engage in crosstalk across several important pathways activated in GBM such as the PI3K/Akt/mTOR, Wnt-β-catenin signaling, MAPK, and Notch signaling pathways. (Figure 3). According to a microarray-mining analysis performed by Zhang and colleagues [113], 129 lncRNAs were differentially expressed between gliomas and normal brain tissues. Han and colleagues identified an even greater number of lncRNAs relevant to GBM using a high throughput screen. They reported 654 lncRNAs that are upregulated and 654 that are downregulated, in GBM in comparison to normal brain tissue [155]. Similarly, an analysis of the RNA-sequencing dataset from TCGA identified 1288 total lncRNAs that had aberrant expression in all gliomas relative to normal brain. The authors found that 584 of these lncRNAs were associated with poor prognosis in GBM, whereas 282 were associated with better survival outcomes [156]. Throughout these studies, certain lncRNAs, including CRNDE (colorectal neoplasia differentially expressed), HOTAIRM1 (HOX antisense intergenic RNA myeloid 1), and MEG3 (maternally expressed 3) were consistently found to be aberrantly expressed in GBM, suggesting that they may be important in gliomagenesis [157]. Some of the lncRNAs that have been extensively studied and are associated with regulating many hallmark parameters of GBM are discussed below (Table 1).

#### 3.2.1. Cell Proliferation and Apoptosis

The CRNDE was initially identified as overexpressed in colorectal cancer but was later found to be elevated in many other cancers as well. It is highly upregulated in gliomas, especially GBM [113]. Knockdown of CRNDE attenuates cell growth and migration in vitro and prevents glioma growth in vivo, suggesting that CRNDE functions as an oncogene [67,136,139]. The CRNDE promotes malignant progression of glioma by acting as a ceRNA sponge for miR-384 and inhibiting the activation of miR-384 target piwi-like RNA-mediated gene silencing 4 (PIWIL4) and its downstream protein signal transducer and activator of transcription 3 (STAT3) [158]. Additionally, CRNDE has been shown to regulate GSC proliferation, migration, and invasion via sponging of miR-186 [159].

MEG3 is a lncRNA that is significantly downregulated in gliomas and is associated with advanced tumour grade, recurrence, IDH wild-type status, and poor overall survival [112]. The overexpression of MEG3 in vitro suppresses proliferation and promotes apoptosis and autophagy in glioma cells [107]. Although the exact mechanism of action of MEG3 in GBM still remains to be elucidated, several miRNAs have been identified with direct binding sites on MEG3, suggesting that it may function as a ceRNA [107,108].

In another study, Hu et al. reported that lncRNA PLAC2 (placenta-specific protein 2) induces cell cycle arrest in glioma through interacting with signal transducer and activator of transcription 1 (STAT1) and targeting ribosomal protein (RP) L36. The authors reported that PLAC2 overexpression inhibits cell proliferation and induces G1/S arrest via modulation of RPL36 expression, and inhibits glioma growth in vivo in a xenograft model [104,105,106].

#### 3.2.2. Migration and Invasion

More recently, lncRNA-ATB (activated by TGF-β) has been shown to promote TGF-β-induced invasion of glioma cells. Overexpression of lncRNA–ATB resulted in activation of the NF-kB pathway and translocation of p65 into the nucleus, thus facilitating glioma cell invasion by TGF-β. Moreover, lncRNA–ATB enhanced TGF-β-mediated invasion of glioma cells through activation of p38/MAPK pathway [110]. Another lncRNA EPIC1 (epigenetically induced lncRNA1) is highly upregulated in glioma and has been reported to modulate several important cellular processes. Its inhibition induces apoptosis and suppresses cell viability, increases cell sensitivity to TMZ, and significantly attenuates cell invasion via targeting of Cdc20 [93].

Similarly, lncRNA PVT1 (plasmacytoma variant translocation 1), was shown to facilitate tumourigenesis and glioma progression via the regulation of bone morphogenetic protein (BMP) signaling pathway. The lncRNA PVT1 acted as a sponge of miR-128-3p, and as a result, influenced the expression of BMP2 and BMP4 by regulating Gremlin 1 (GREM1). Knockdown of lncRNA PVT1 attenuated glioma cell proliferation, invasion, and migration in vitro and produced smaller tumours in a mouse model [100].

#### 3.2.3. Angiogenesis

H19 imprinted maternally expressed transcript (H19), one of the first lncRNAs to be identified, is also upregulated in GBM. H19 had previously been shown to be induced in GBM by c-Myc [101]. Further reports suggested that H19 may act as a ceRNA to regulate HIF-1α through miR-138 sponging, and thus promote angiogenesis in GBM [160]. Another ceRNA, CCAT-1, is also upregulated in gliomas. CCAT-1 sponges miR-181b and regulates the de-repression of endogenous targets fibroblast growth factor receptor 3 (FGFR3) and PDGFR-α, thus promoting glioma tumourigenesis. Moreover, knockdown of CCAT-1 promotes apoptosis and suppresses proliferation, migration, and EMT transition of glioma cells [97].

#### 3.2.4. Therapeutic Resistance

LncRNAs have also been associated with predicting therapeutic response in GBM patients. Garcia-Claver and colleagues [92] analyzed the expression profiles of human glioma cell lines to identify gene expression changes associated with erlotinib (ERL) treatment. One of the genes they reported significantly upregulated after treatment in both ERL-resistant and ERL-sensitive glioma cell lines was the lncRNA growth arrest specific 5 (Gas5). In addition, the knockdown of Gas5 resulted in increased sensitization of GBM cells to erlotinib [96]. Similarly, lncRNA small nucleolar RNA host gene 12 (SNHG12) was highly upregulated in TMZ-resistant cells. SNHG12 regulates the G1/S cell cycle transition by competitively binding to miR-129-5p, and upregulates MAPK1 and E2F7, thus leading to TMZ resistance in GBM cells [109].

In a CRISPR (clustered regularly interspaced short palindromic repeats) based radiation modifier screen, Liu et al. [98] identified nine lncRNAs that sensitized glioma cells to radiation. Additionally, they found that lncRNA Glioma Radiation Sensitizer 1 (lncGRS-1) selectively decreased tumour growth in a human brain organoid model and sensitized glioma cells to radiation. In a similar manner, Mazor et al. [111] found that TP73-AS1 was overexpressed in primary GBM and can serve as a prognostic biomarker. Additionally, the inhibition of TP73-AS1 results in loss of ALDH1A1 expression, which re-sensitizes GSCs to TMZ treatment.

#### 3.2.5. Self-Renewal

Another lncRNA that is highly upregulated in gliomas and is associated with poor patient survival outcomes is HOTAIRM1. It is an enhancer lncRNA (lnc-eRNA) that promotes glioma cell proliferation by regulating long-range chromatin interactions within HOXA cluster genes [96]. HOTAIRM1 has been shown to maintain GSC proliferation, apoptosis, self-renewal, and turmorigencity by regulating the HOXA2 and HOXA3 gene expression [161]. Another report suggested that the knockdown of HOTAIRM1 suppresses the malignant behavior of gliomas and increases tumour cell sensitivity to TMZ. The authors suggested that HOTAIRM1 promotes malignancy of gliomas by acting as a sponge for miR-129-5p and miR-495-3p [99].

Our lab has recently identified identify a novel lncRNA, Cancer stem cell associated distal enhancer of SOX2 (CASCADES) that functions as an epigenetic regulator in GSCs. CASCADES is expressed in IDH wild-type GBM and significantly enriched in GSCs Knockdown of CASCADES in GSCs results in a decrease in the stemness markers, Nestin and Sox2, an increase in the neuronal marker, Tuj1, and a corresponding decrease in Cyclin B1 and Olig2, consistent with differentiation of GSCs towards a neuronal lineage in a cell- and cancer-specific manner. Bioinformatics analysis reveals that CASCADES is a super-enhancer-associated lncRNA and functions as an epigenetic regulator of SOX2. Our results show that *CASCADES* represents an exciting epigenetic target for disrupting the GSC niche (Shahzad et al., unpublished; doi:10.1101/2020.09.05.284349).

Without a doubt, lncRNAs are emerging as important entities in cancer development and progression by acting through a variety of cellular and physiological regulatory mechanisms. We have barely begun to scratch the surface of the potential of lncRNAs as molecular targets in GBM. Further research is needed to fully exploit the diagnostic and therapeutic potential of lncRNAs in GBM and other cancers.

## 4. Circular RNAs—Emerging Class of Genetic Regulators

**Figure 4:**
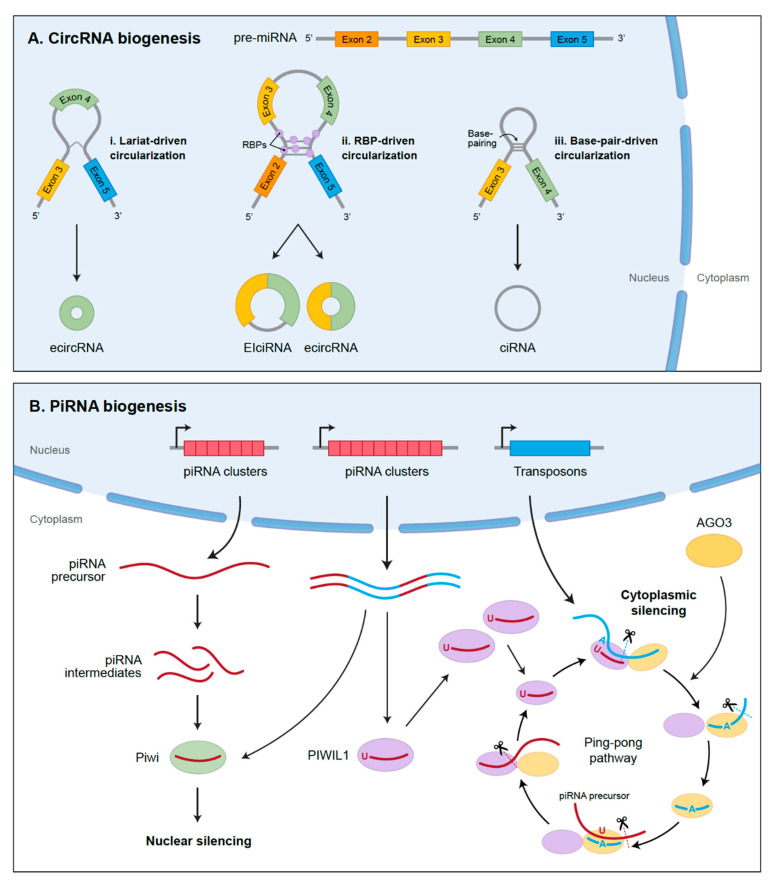
Biogenesis of circRNA and piRNA. (**A**) CircRNAs are generated from pre-mRNA through back-splicing, connecting 5’ splice site to an upstream 3’ slice site. The exon circRNAs (ecircRNAs) are synthesized by either RNA-binding protein (RBP)-driven circularization or lariat-driven circularization that involves exon skipping due to partial folding of the RNA during transcription of the pre-mRNA. Exon intron circRNAs (EIciRNAs) retain the introns during biogenesis, whereas ciRNAs are derived from lariat intron that is excised from pre-mRNA. (**B**) PiRNAs are transcribed from piRNA clusters and are processed from single-stranded precursor transcripts. They are then loaded onto Piwi or PIWIL1 (or its isoforms PIWIL2, PIWIL3, and PIWIL4). Alternatively, they are amplified through the Ping-Pong cycle, in which PIWI proteins associated with antisense piRNAs cleaves piRNA precursors in the sense strand, or vice versa. The Ping-Pong pathway silences the expression of the target transposon, while simultaneously amplifying the piRNA sequence.

Circular RNAs are a class of noncoding RNAs that are covalently closed, single-stranded transcripts, which lack 5’ caps and 3’ poly(A) tails (Figure 4A). They are generated from precursor mRNA (pre-mRNA) through back-splicing, connecting a downstream splice donor site (5’ splice site) to an upstream acceptor splice site (3’ slice site) [162]. CircRNAs generally have low expression levels and have tissue-specific functions [163,164,165]. Because of their closed loop structure, they are resistant to degradation by ribonucleases and are relatively stable compared to mRNAs [166,167,168,169]. Circular RNAs were originally discovered in 1976 by Sanger and colleagues, who discovered a single-stranded RNA molecule responsible for potato spindle tuber disease that resembled a viroid but lacked a protein envelope [163,164,165]. In the early 1990s, the presence of circRNAs in higher eukaryotes was confirmed [170]. While they were previously thought to act as “splicing noise” with little functional potential, recent studies have demonstrated that circRNAs are abundant molecules in the brain and have significant biological functions, especially in cancer.

Circular RNAs are classified into three categories: exon circRNAs (ecircRNAs), circular intron RNAs (ciRNAs), and exon intron circRNAs (EIciRNAs) [171,172,173]. The ecircRNAs are synthesized by either RNA-binding protein (RBP)-driven circularization, lariat-driven circularization that involves exon skipping due to the fact of partial folding of the RNA during transcription of the pre-mRNA, or intron-pairing-driven cyclization that relies on a reverse shear mechanism forcing two flanking introns to pass through the ALU complementary component. Exon intron circRNAs are considered an intermediate product from the biogenesis of ecircRNAs and have stable retention of introns between exons. Circular RNAs are derived from lariat intron excised from pre-mRNA by canonical splicing machinery and are characterized by a 7-nucleotide GU-rich motif near the 5’ splice site and 11 nucleotides near the C-containing motif, which makes them resistant to the effects of debranching enzymes [167].

Circular RNAs can have a variety of functions including miRNA sponging, transcriptional regulation by competing with the linear splicing of pre-mRNA to modulate the expression of related genes, and binding to proteins to act as decoys. Some newer models have suggested that circRNAs can be also translated [164,174,175,176,177]. Circular RNAs have also been implicated in glioma progression and pathogenesis (Table 2). In a study analyzing the RNA-seq data from 46 gliomas and normal brain tissues, more than 476 circRNAs were found to be differentially expressed in gliomas [176,178,179,180,181]. cir-ITCH is a circRNA that is downregulated in gliomas and can be considered a prognostic biomarker. Under normal physiologic circumstances, cir-ITCH positively regulates the expression of tumour suppressor gene ITCH (itchy E3 ubiquitin protein ligase) and promotes the expression of ITCH by sponging miR-214 and suppressing Wnt/β-catenin signaling and inhibiting cell proliferation [182].

Zheng et al. reported that circ-TTBK2, but not linearized TTBK2, was upregulated in gliomas, and was associated with enhanced cell proliferation, migration, and invasion. Additionally, it inhibited apoptosis by acting as a miR-217 sponge and regulating the HNF1β/Derlin-1 pathway [185,186]. Another important circRNA is cZNF292, which is expressed during hypoxia and has been shown to inhibit glioma cell proliferation, tube formation, as well as S/G2/M phase cell cycle arrest by regulating the Wnt/β-catenin pathway [194].

Circular RNAs are abundantly expressed in the human brain. It is becoming increasingly evident that they are important in the process of glioma development and progression. Enhanced understanding of these biological entities and glioma pathogenesis will be necessary for the potential clinical application of circRNAs in the diagnosis and treatment of this tumour.

## 5. Piwi-Interacting RNAs—Small RNAs with Big Functions

Piwi-Interacting RNAs were originally identified in 2001 by Aravin and colleagues [184] in *Drosophila melanogaster* as a class of “long siRNAs” that silence *Stellate*, a multi-copy gene on the X chromosome. They are quite possibly the largest class of noncoding RNAs in mammals, with over 30,000 members identified in humans alone, mapping to thousands of retrotransposable element-encoding genomic loci, and have been associated with many functional and disease processes in mammals, including cancer development [199].

Piwi-Interacting RNAs are 25–27 long nucleotides that are transcribed from genomic loci known as piRNA clusters and are processed from long single-stranded precursor transcripts, and then loaded onto Piwi family of proteins (Figure 4B). Alternatively, they are amplified through the Ping-Pong cycle, in which PIWI proteins associated with antisense piRNAs cleaves piRNA precursors in the sense strand, or vice versa [200,201,202,203,204,205]. The biological functions of piRNA depend on their cellular location, but primarily involve silencing of transposons. Transposons are regions of the genome that can change their positions within the genome, and can promote illegitimate recombination, double-stranded DNA breaks, or disruption of coding sequences by insertion into new sites [190,193,194]. In the nucleus, piRNA–Piwi complexes can repress transposon expression by methylating transposon regions or introducing chromatin modifications around transposons. In the cytoplasm, however, piRNAs can degrade retrotransposon-associated mRNAs, facilitate mRNA maturation, and promote cleavage of mRNAs through miRNA-like mechanisms [206,207,208,209].

In cancer specifically, piRNA–Piwi complexes have been shown to mediate transcriptional gene silencing through sequence complementarity and recruitment of silencing machinery components, as well as post-transcriptional gene silencing through piRNA–RNA interactions similar to miRNA mechanisms [197,210,211,212]. In addition to this, piRNA–Piwi complex can also recruit DNA methyltransferase (DNMT) to methylate CpG islands located near the non-transposable element sites within the genome, effectively altering transcriptional activity [210]. Finally, the piRNA–Piwi complex can directly bind to proteins and facilitate their activation through phosphorylation, thereby promoting signal pathway activation [213,214].

While piRNAs have been implicated in other cancers, the literature exploring their role in gliomagenesis is limited (Table 2). The PIWI family gene, PIWIL1, has been shown to regulate growth, invasion, and migration of glioma cells in both in vitro and in vivo glioma models, and has been associated with poor prognosis [215]. In 2016, Jacobs et al. collected the data on 1840 glioma patient samples and 2401 controls from the GliomaScan genome-wide association study and analyzed the association between 1428 piRNAs and gliomas. They identified five piRNAs that were associated with increased glioma risk. Additionally, piR-598 was found to enhance glioma cell survival and colony formation [216,217]. In addition, downregulation of PIWIL1/piRNA-DQ593109 (piR-DQ593109) has been shown promote blood tumour barrier (BTB) permeability through the MEG3/miR-330-5p/RUNX3 axis [195]. In another study, PIWIL3/OIP5-AS1/miR-367-3p/CEBPA (CCAAT/enhancer binding protein alpha) feedback loop was found to regulate glioma cell growth, and the overexpression of PIWIL3 or piR-30188, either jointly or separately suppressed glioma progression [218].

The field of piRNA biology is relatively new but shows promise due to the ability of piRNAs to regulate gene expression via epigenetic mechanisms. Further research may elucidate their role in regulating tumorigenesis and their potential as therapeutic and prognostic markers.

## 6. Therapeutic Targeting of Noncoding RNAs

### 6.1. Current Technologies for Translational Application

**Figure 5:**
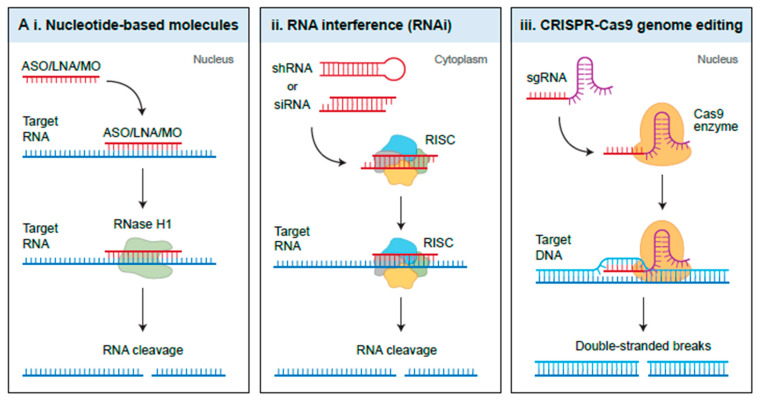

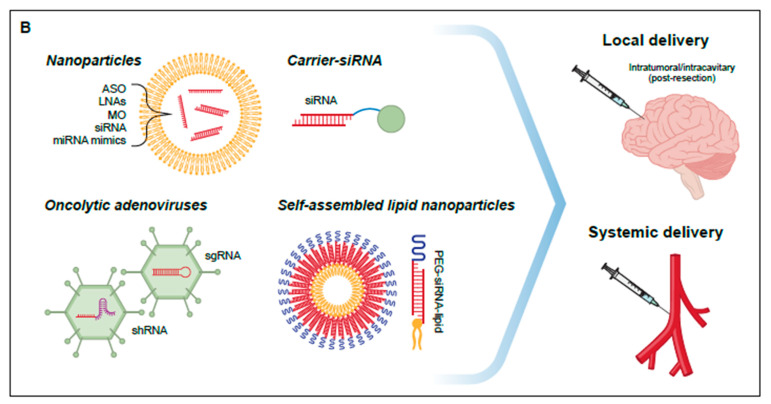
Therapeutic targeting of NcRNAs. (**A**) Schematic of the three common methods used to target ncRNAs. i. Nucleotide-based molecules, such as antisense oligonucleotides (ASOs), locked nucleic acids (LNAs), and morpholino oligonucleotides (MO) bind to their targeted RNA and use endogenous RNase H1 to promote RNA cleavage. ii. RNA interference (RNAi) involves the use of short interfering RNAs (siRNAs) and short hairpin RNAs (shRNAs) and utilizes the RNAi-induced silencing complex (RISC) to specifically degrade the targeted RNA. iii. CRISPR-Cas9 editing makes alterations at the genomic level by using a target specific single-guide RNA (sgRNA) and the Cas9 nuclease that specifically cleaves the genomic locus. (**B**) There are two possible delivery methods to deliver the ncRNA therapeutics: either local delivery within the tumour, or systemic. However, one of the main challenges with systemic delivery is overcoming the blood–brain barrier. To enhance their delivery, nucleotide-based therapeutics and siRNAs can be encapsulated inside nanoparticles. SiRNAs can also be delivered by chemically conjugating with carrier molecules, or through self-assembled lipid nanoparticles that are modified with PEG. ShRNAs and sgRNAs can be delivered by utilizing oncolytic adenoviruses.

Undoubtedly, several ncRNAs have been shown to play important roles in regulating glioma biology and can serve as potential therapeutic targets or biomarkers. Some of the emerging technologies to target noncoding RNAs include antisense oligonucleotides (ASOs), locked nucleic acids (LNAs), peptide nucleic acids (PNAs), and morpholino oligonucleotides (MO) (Figure 5A) [197].

ASOs are single-stranded oligonucleotides that have complementary sequences to the target RNAs and inhibit their activity by direct binding and promoting their degradation by RNase H. They can be used to target mRNAs, miRNAs, lncRNAs, circRNAs, and piRNAs, and can be delivered naked without the use of a vehicle [219,220]. ASOs have been shown to effectively inhibit the expression of lncRNA MALAT1 and attenuate cancer cell metastasis and tumour burden in mice [221,222,223]. In addition to this, Teplyuk and colleagues used ASO to inhibit miR-10b in human GSC-derived xenografts and murine GL261 allograft models. The miR-10b ASO was delivered through direct intratumoral injections, continuous osmotic delivery, and systemic intravenous injections. The authors reported that all three delivery methods proved efficient in achieving ASO-mediated miR-10b inhibition with minimal toxicity, leading to the de-repression of target RNAs and attenuated tumor growth and progression [224].

Locked nucleic acids are single-stranded oligonucleotides containing stretch of DNA flanked by bicyclic nucleotides with locked conformation, generating a chimeric molecule that is strongly resistant to nuclease degradation. LNA oligonucleotides have been shown to inhibit antiapoptotic activities of miR-21 in GBM cell lines [225]. In a study by Griveau and colleagues, the authors successfully silenced miR-21 in U87MG GBM cells by using LNA conjugated to lipid nanocapsules (LNCs) and demonstrated increased sensitivity of GBM cells to radiation-induced cell death [70]. Moreover, Wang et al. used a LNA to silence miR-381 in U251 GBM cells and increased sensitivity to TMZ [226]. Similarly, PNAs are stretches of nucleic acid sequences flanked by peptides to increase target affinity, specificity, resistance, and penetration [227]. PNAs designed with four lysine residues have been shown to inhibit miR-155 function in mice in vivo [220,228].

Morpholinos are 25-nt non-ionic DNA analogs that hybridize to the cognate site of their target RNA and promote RNase H-mediated degradation. In a study by Lu et al., MO delivered via nanoparticles to target a MYC-inducible lncRNA DANCR in a xenograft model of human ovarian cancer demonstrated strong suppression of tumour growth [229]. Salphati and colleagues used GDC-0084, a morpholino to target phosphatidylinositol 3-kinase (PI3K) with modifications to cross the blood-brain barrier (BBB), in an orthotopic xenograft model of GBM. GDC-0084 markedly inhibited the PI3K pathway in mouse brain, and significantly inhibited tumor growth. Furthermore, the matrix-assisted laser desorption ionization (MALDI) imaging of xenografts showed an even distribution of the drug in the brain and intracranial tumors [230].

In addition to MO, both miRNA mimics and antagomirs have been used to target miRNAs. As the name suggests, antagomirs are 22–23 nucleotide long RNA analogs that are used to knockdown the expression of miRNAs. Antagomirs are nuclease resistant and can be delivered to cells directly without the use of any vectors or delivery vehicles [231]. In contrast, miRNA mimics are an approach to overexpress the target miRNA by synthesizing an oligonucleotide with a sequence similar to the mature endogenous miRNA. For example, in a study by Sun et al., miR-137 mimics were shown to negatively regulate neural stem cell proliferation [219,228].

In addition to nucleotide-based molecules to target noncoding RNAs, small ncRNAs have also been used as potential therapeutic tools. Short interfering RNAs (siRNAs) are a class of short double-stranded RNAs that complementarily bind to their target RNA, including mRNA, lncRNA, and circRNA, and silence their expression in a RISC dependent manner [232]. Similarly, short hairpin RNAs (shRNAs) have been widely utilized to target a variety of noncoding RNAs and inhibit their expression both in vitro and in vivo [233]. Additionally, the discovery of CRISPR-associated (Cas) system has made precise gene editing possible, and this technology can be harnessed as a therapeutic tool for targeting ncRNAs. The CRISPR-Cas9 system involves the use of a 20-nucleotide single-guide RNA (sgRNA) designed against a target genomic sequence, and the Cas9 nuclease that specifically cleaves the genomic locus [234]. In a study by Peng and colleagues, CRISPR-Cas9 was employed to successfully knockout linc-RoR in breast cancer cell lines, and as a result, attenuated estrogen deprivation-induced ERK activation [235,236].

Besides nucleotide-based and RNAi-based approaches, small molecules can be used to therapeutically target ncRNAs, depending on their structural features. High throughput screening methods, such as small-molecule microarrays have been used to identify small molecules that bind to several structured forms of RNA [237]. In a small, targeted screen performed in MCF-7 cells, Bose et al. identified streptomycin as a highly potent miR-21 inhibitor, with level of inhibition comparable to that of an ASO designed against miR-21 [238,239,240,241].

More recently, the chemoinformatics approach called Inforna, developed by Disney and colleagues, has been used to identify many lead compounds for targeting RNA [242]. This strategy involves using a database of RNA motif and small molecule interactions that have been experimentally verified through 2-dimensional combinatorial screening (2DCS) and a binding fitness score based on structure activity relationships through sequencing (StARTS). Therefore, Inforna can predict targetable structural motifs of any RNA, including ncRNA, and corresponding small molecule hits, as well as the fitness of their interaction [241,243]. Using this approach, G Neomycin B (G Neo B) was identified as a small molecule drug, which inhibited the production of mature miR-10b through direct binding to Drosha binding site [243,244,245].

The rapid evolution of nucleotide-based and non-nucleotide-based technologies in the recent years has offered a variety of options to target ncRNAs therapeutically in the clinic. The fact remains that most of these approaches have been investigated only in the preclinical stage for GBM, and additional studies are needed to fully explore their potential for translational application.

### 6.2. From “Bench-to-Bedside”—NcRNA Therapies and Their Road to the Clinic

Since the field of ncRNA biology is relatively new, there are limited ncRNA-based therapies already approved for use in the clinic for GBM, or in fact, many other cancers. However, several clinical trials are currently underway to investigate the safety and efficacy of ncRNA-based theranostics. A well-known example of a ncRNA that has been approved by the U.S. Food and Drug Administration (FDA) for diagnostic purposes is prostate cancer antigen 3 (PCA3) [246]. PCA3 is a prostate-specific lncRNA that is highly overexpressed in most types of prostate cancer cells and is indicated for detecting the presence of malignancy in men undergoing repeat prostate biopsy. Unlike serum prostate specific antigen (PSA) levels, PCA3 is not affected by the prostate volume or non-cancerous prostate hyperplasia, making it a particularly useful biomarker, especially for identifying patients with clinically insignificant prostate cancer [247,248,249,250,251]. The successful case study of PCA3 illustrates that ncRNAs, which usually have tissue-specific and cell-specific expression patterns, can be utilized as important diagnostic or prognostic biomarkers in many cancers, including GBM.

In a similar manner, lncRNA HOTAIR is currently being investigated as a putative biomarker for thyroid cancer in an observational study (ClinicalTrials.gov Identifier: NCT03469544). Likewise, miR-221 and miR-222 are being studied as clinical biomarkers for hepatocellular carcinoma (ClinicalTrials.gov Identifier: NCT02928627). Moreover, a prospective observational study is currently recruiting to evaluate miR-10b as a prognostic and diagnostic biomarker of various glioma subclasses (ClinicalTrials.gov Identifier: NCT01849952).

In addition, nucleotide-based therapeutics to target both ncRNAs and mRNAs of several genes have been investigated in clinical trials and have shown great promise, further providing important information for improving their use in the clinic. In a phase IIa clinical trial, a miR-122 inhibitor named Miravirsen showed a dose-dependent reduction in the hepatitis C virus (HCV) RNA levels in patients with chronic HCV genotype 1 infection [249,251]. Miravirsen is an LNA-based oligonucleotide complementary to miR-122 and leads to long-lasting suppression of HCV viremia by derepressing target mRNAs with miR-122 seed sites, as well as downregulating interferon-regulated genes, and improving overall liver pathology [252]. Similarly, a miRNA-mimic, MRX34, has also entered clinical testing. MRX34 is a miR-34a mimic that is encapsulated in a liposomal nanoparticle (SMARTICLES), and has been shown to inhibit human hepatocellular carcinoma cells, leading to tumor regression and prolonged survival in mouse models [253,254]. In a phase I trial, MRX34 was administered to nonhuman primates to determine its pharmacokinetics [255,256]. Following this, a clinical trial was designed to study the pharmacodynamics of MRX34 in melanoma patients (ClinicalTrials.gov Identifier: NCT02862145). At the time of the publication of this paper, this clinical trial is listed as “withdrawn” due to the serious immune-related adverse events. It is unclear whether the adverse effects were due to the miRNA-mimic or the delivery system.

An ASO-based therapeutic, nusinersen, was approved by the FDA in 2016 to treat spinal muscular atrophy (SMA). Nusinersen is a steric block ASO that blocks an intronic splice suppressor element found in SMN2 (of motor neuron 2) and, therefore, promotes full-length SMN expression [257]. In a phase II clinical trial, intrathecal administration of nusinersen showed improvements in motor function for patients who received high doses of the drug. Moreover, post-mortem analysis of the tissue indicated that intrathecal administration of nusinersen led to broad distribution of the drug throughout the spinal cord and the brain, including neurons [258,259,260]. Furthermore, phase III clinical trial in patients with infantile-onset SMA showed marked improvement in motor function and reduced risk of death or permanent ventilation, which led to the approval of nusinersen by the FDA in the USA as well as the European Medicines Agency [261].

Another ASO-based drug designed to target mRNA of oncogenic KRAS (Kirsten rat sarcoma), AZD4785, has entered clinical trials. KRAS is frequently amplified in GBM [221,262], and previous attempts to enzymatically inhibit it have been largely unsuccessful. However, the ASO-based inhibition of KRAS resulted in robust target knockdown in both mice and monkeys without any adverse effects [6,263]. A phase I dose-escalation study of AZD4785 in patients with non-small cell lung cancer (NSCLC) has just completed recruitment (ClinicalTrials.gov Identifier: NCT03101839).

In an open-label phase I/IIa study, an siRNA drug against the G12D mutant of KRAS, siG12D-LODER, was delivered for four months to patients with non-operable pancreatic cancer via a miniature biodegradable implant [264]. The results of the trial showed that the combination of chemotherapy and siG12D-LODER was well-tolerated and slowed tumor progression [265]. Overall, the results from the early clinical trials underscore that nucleotide- and RNAi-based therapeutics are generally safe and well tolerated in humans and can be improved upon to achieve desired clinical efficacy.

### 6.3. Challenges in Developing Targeted ncRNA Therapies

Although the many options for targeting ncRNAs are promising, their efficacy is limited by concerns of off-target effects, low transfection efficiency, short half-life, bioavailability, as well as overcoming the BBB. To overcome such challenges, oligonucleotides and morpholinos can be modified chemically to improve their stability and uptake within the cells (Figure 5B) [265]. Additionally, to enhance their delivery, siRNAs, ASOs, and miRNA mimics can be encapsulated inside lipid-based or polymeric nanoparticles. For example, in a study of hepatocellular carcinoma (HCC), Huang et al. delivered VEGF siRNA using lipid/calcium/phosphate nanoparticles conjugated with galactoside derivatives and demonstrated a superior siRNA delivery into HCC cells compared to normal hepatocytes. Additionally, the VEGF expression was significantly downregulated in HCC cells both in vitro and in vivo in a murine orthotopic HCC model [209,210,218].

To improve the durability and precision of small ncRNAs to effectively target their gene of interest, small ncRNAs can be conjugated with siRNA carriers [266]. Nair and colleagues tested this strategy to precisely target hepatocytes by conjugating a siRNA to *N*-acetylgalactosamine (GalNAc). GalNAc has high binding affinity to asialoglycoprotein receptor (ASGPR), which is specifically localized to the surface of hepatocytes. The subcutaneous administration of siRNA-GalNAc conjugates in mice resulted in a sustained dose-dependent gene silencing response in the liver for over 9 months, with no adverse effects [209,210,218]. Another variation of this delivery strategy involves modification with lipid and polyethylene glycol (PEG) molecules, and the use of self-assembled lipid nanoparticles [267]. Yoon et al. used the self-assembled micelle interfering RNA (SAMiRNA) nanoparticles to target amphiregulin (AR) or connective tissue growth factor (CTGF) in TGF-β transgenic mouse models of pulmonary fibrosis. SAMiRNA nanoparticles comprised of individually biconjugated siRNAs with a hydrophilic polymer and lipid on their ends. In vivo delivery of either AR or CTGF SAMiRNA nanoparticles via intratracheal or intravenous injection effectively silenced the expression of target genes, significantly reduced TGF-β-stimulated collagen accumulation in the lung, and substantially restored lung function in mice. Additionally, the use of SAMiRNAs did not induce any toxicity or significant innate immune response [209,210,218]. This suggests that siRNA conjugation with carrier molecules or self-assembling lipid nanoparticles is a safe and effective method to improve the precision and localization to achieve stable siRNA-mediated gene silencing.

Lastly, oncolytic adenoviral vectors can be used to deliver small ncRNAs, including sgRNAs [268]. As an example, Machitani and colleagues generated a telomerase-specific replication-competent adenovirus (TRAD), which expressed shRNA designed against Dicer (shDicer). The TRAD carried the human telomerase reverse transcriptase (hTERT) promoter-driven E1 gene expression cassette and exhibited antitumor activity and higher replication efficiency. After transfection of HeLa cells and other various cancer cell lines including HCC, ovarian carcinoma, NSCLC cells with TRAD expressing shDicer, the Dicer expression levels were selectively inhibited in tumor cells [209,210,218]. These results indicate that oncolytic adenoviral vectors are effective transport vehicles to deliver small ncRNAs designed against specific genes-of-interest and can be used to selectively target tumor cells.

While all of these delivery strategies have their limitations, they are continually being improved to enhance the bioavailability of these nucleic acid drugs for potential translational applications. A major challenge remains that there are only a handful of studies, both preclinical and clinical, which thoroughly investigate the different strategies to target ncRNAs in GBM. However, the lessons learned from therapeutic targeting of ncRNAs in other diseases can be extrapolated to GBM and help inform future research that aims to improve these potential strategies and make targeted therapeutics a reality by bringing them to the clinic with the overall goal of improving patient outcomes.

## 7. Conclusions and Future Directions

Noncoding RNAs have emerged as a novel class of biological regulators and have a variety of different functions in modulating tumorigenesis. With the advent of sequencing technology, several different classes of ncRNAs have been uncovered, including miRNAs, lncRNAs, circRNAs, and piRNAs, all of which are potential players in glioma biology. They are frequently dysregulated in GBM and regulate many aspects of glioma development including cell proliferation, migration, invasion, apoptosis, angiogenesis, and self-renewal. Due to the diverse nature of their biogenesis, length, and mechanisms, the development of more streamlined approaches is needed to help understand their dynamics as potential therapeutic targets within the context of GBM. Moreover, since many ncRNAs have tissue-specific expressions, they can be investigated as potential predictive and prognostic biomarkers to predict treatment outcomes as well as resistance to chemoradiation. Future research should also focus on understanding the role of other class of ncRNAs, including snoRNAs and tRNAs, in glioma development.

In addition, nucleic acid therapeutics, including ASOs, LNAs, and MOs, can be used to target ncRNAs and modulate their expression both in vitro and in vivo. The number of approaches to modify these therapeutics and improve their delivery and bioavailability is making therapeutic targeting of ncRNAs in the clinic a reality. However, better understanding of the off-target effects of nucleic acid therapeutics and potential toxicity is needed. CRISPR-Cas9 is another exciting technology that can be used to target ncRNAs, however, further research is needed to fully understand its effects and application.

In conclusion, ncRNAs represent an exciting class of biomolecules for regulating many hallmark characteristics of GBM and can be exploited to better manage the progression of this disease.

## Figures and Tables

**Table 1:** Key functional miRNAs and lncRNAs in glioblastoma (GBM).

ncRNA	Expression Change in GBM	Role	Reference
*miRNA*			
miR-10b	Increase	Promotes TMZ-resistance, proliferation, migration, invasion, and stemness	[64]
miR-17-92 cluster	Increase	Regulates glioma cancer stem cell (GSC) differentiation, apoptosis, and proliferation	[65,66,67,68]
miR-21	Increase	Targets tumour suppressor genes like PDC4, AANP32A, SMARCa4, PTEN, and SPRY2. Inhibition leads to reduced cell proliferation and tumour growth and enhanced sensitivity to chemoradiation	[69]
miR-34a	Decrease	Inhibits expression of MET, NOTCH1/2, CDK6, CCND1, and SIRT1	[62,65,70,71,72,73,74,75,76,77,78]
miR-93	Increase	Suppresses integrin-β8 and enhances cell survival, sphere formation, and blood vessel formation	[79,80,81]
miR-125b	Decrease	Targets MAZ. Knockdown promotes tumour vascularization	[82]
miR-146b	Increase	Inhibits MMP16 and enhances cell invasion	[83]
miR-195	Increase	Promotes TMZ resistance	[84]
miR-218	Decrease	Targets HIF-2α and attenuates tumour vascularization and prevents cell survival	[85]
miR-221/222	Increase	Targets tumour suppressor p27 and PUMA. Overexpression inhibits apoptosis and promotes cell survival	[86]
miR-296	Increase	Increases endothelial cell tube formation and enhances vascularization of tumours	[87,88,89,90]
*lncRNA*			
CCAT-1	Increase	Sponges miR-181b and promotes proliferation, migration, and EMT transition	[91]
CRNDE	Increase	Acts as an oncogene, and regulates proliferation, migration, invasion, and stemness	[92]
EPIC1	Increase	Inhibition suppresses cell viability, induces apoptosis, and increased ell sensitivity via targeting of CDC20 in glioma cells	[93]
GAS5	Increase	Promotes tumour cell resistance to erlotinib	[94,95]
H19	Increase	Acts as a ceRNA for miR-138 and regulates HIF-1α, promoting angiogenesis	[96]
HOTAIRM1	Increase	Regulates long-range chromatin interactions within HOXA cluster genes, and maintains GSC proliferation, apoptosis, and self-renewal	[97]
lncGRS-1	Increase	Knockdown inhibits the growth of glioma cells	[98]
lncRNA-ATB	Increase	Promotes TGF-β induced invasion of glioma cells through activation of p38/MAPK	[99]
lncRNA PVT1	Increase	Acts as a sponge for miR-128-3p, and promotes glioma cell proliferation, invasion, and migration	[100]
MALAT1	Increase	Induces chemoresistance to temozolomide. Correlated with poor prognosis	[101]
MEG3	Decrease	Regulates proliferation, apoptosis by potentially acting as a ceRNA for miRNAs	[102,103]
PLAC2	Decrease	Induces cell cycle arrest in glioma through interaction with STAT1 and RPL36	[104,105,106,107,108]
SNHG12	Increase	Promotes temozolomide (TMZ) resistance in GBM cells. Serves as a sponge for miR-129-5pp, leading to upregulation of MAPK1 and E2F7	[109]
SOX2OT	Increase	Regulates GSCs through miR-194-5p and miR-122	[110]
TP73-AS1	Increase	Overexpressed in GSCs. Promotes TMZ resistance by regulating the expression of ALDH1A1	[111]
XIST	Increase	Promotes glioma tumorigenicity and angiogenesis by sponging miR-429. Maintains GSCs via miR-152	[112]

**Table 2:** Key functional circular RNAs (circRNAs) and piRNAs in GBM.

ncRNA	Expression Change in GBM	Role	Reference
*circRNA*			
cZNF292	Decrease	Regulates Wnt/B-catenin pathway. Inhibits glioma cell proliferation and tube formation	[183]
cir-ITCH	Decrease	Prognostic biomarker. Promotes expression of ITCH by sponging miR-214 and suppressing Wnt/B-catenin	[184]
circBRAF	Decrease	Negatively correlates with tumour malignancy grade	[185,186]
circFBXW7	Decrease	Inhibits proliferation and cell cycle of glioma cells	[187]
circNFIX	Increase	Sponges miR-34a-5p and regulates Notch signaling	[188,189]
circNT5E	Increase	Sponges miR-422a and regulates cell proliferation, migration, and invasion	[190]
circSHKBP1	Increase	Interacts with miR-544a and miR-379 to regulate angiogenesis	[191]
circSMARCA5	Decrease	Inhibits glioma cell migration	[192]
circTTBK2	Increase	Associated with enhanced cell proliferation, migration, and invasion. Sponges miR-217	[193]
*piRNA*			
piR-598	Polymorphism	Enhances glioma cell survival and colony formation	[194]
piR-8041	Decrease	Suppresses tumour growth	[195]
piR-30188	Decrease	Involved in PIWIL3/OIP5-AS1/miR-367-3p/CEBPA feedback loop. Overexpression leads to suppressed glioma progression	[196]
piR-DQ590027	Decrease	Regulates the permeability of glioma conditioned normal BBB	[197]
piR-DQ593109	Increase	Downregulation promotes blood tumour barrier permeability	[198]
